# Could A Deletion in Neuraminidase Stalk Strengthen Human Tropism of the Novel Avian Influenza Virus H7N9 in China, 2013?

**DOI:** 10.3390/ijerph120101020

**Published:** 2015-01-20

**Authors:** Liang Chen, Feng Zhu, Chenglong Xiong, Zhijie Zhang, Lufang Jiang, Yue Chen, Genming Zhao, Qingwu Jiang

**Affiliations:** 1 Department of Sanitary Microbiology, School of Public Health, Fudan University, Shanghai 200032, China; E-Mails: chenliangfd@sina.com (L.C.); jianglufang@fudan.edu.cn (L.J.); 2 Key Laboratory of Public Health Safety, Ministry of Education, Fudan University, Shanghai 200032, China; E-Mails: epistat@gmail.com (Z.Z.); gmzhao@shmu.edu.cn (G.Z.); xiongchenglong1@sina.com (Q.J.); 3 Intensive Care Unit of Burn and Trauma Center, Changhai Hospital, Second Military Medical University, Shanghai 200433, China; E-Mail: z8160720@126.com; 4 Department of Epidemiology and Biostatistics, School of Public Health, Fudan University, Shanghai 200032, China; 5 Department of Epidemiology and Community Medicine, Faculty of Medicine, University of Ottawa, Ontario K1H 8M5, Canada; E-Mail: ychen@uottawa.ca

**Keywords:** deletion, neuraminidase, H7N9, China

## Abstract

*Objective.* A novel avian influenza A virus (AIV) H7N9 subtype which emerged in China in 2013 caused worldwide concern. Deletion of amino-acids 69 to 73 in the neuraminidase stalk was its most notable characteristic. This study is aimed to discuss the tropism and virulence effects of this deletion. *Methods*: Neuraminidase gene sequences of N9 subtype were collected from NCBI and GISAID. MEGA6.0, Stata12.0, and UCSF Chimera were employed for sequence aligning, significance testing, and protein tertiary structure homology modeling. *Results*: A total of 736 sequences were obtained; there were 81 human isolates of the novel AIV H7N9, of which 79 had the deletion. Among all the 654 avian origin sequences, only 43 had the deletion (*p* < 0.001). Tertiary structure displayed that the deletion obviously changed the spatial direction of neuraminidase. *Conclusions*: The deletion in neuraminidase stalk could have strengthened human tropism of the novel AIV H7N9, as well as its virulence.

## 1. Introduction

A novel avian influenza A virus (AIV) H7N9 subtype was detected in China on 19 February 2013, which caused worldwide concern [1,2]. As of 11 April 2014, the virus had spread to ten provinces (Anhui, Fujian, Guangdong, Hebei, Henan, Hunan, Jiangsu, Jiangxi, Shandong, and Zhejiang), two metropolitan cities (Shanghai and Beijing), and Hong Kong. Of 398 patients with H7N9 influenza, 77 (19.35%) died, and the fatality rate was much higher compared to that of the pandemic influenza H1N1 in 2009–2010 (<0.25%) [3,4].

**Figure 1:**
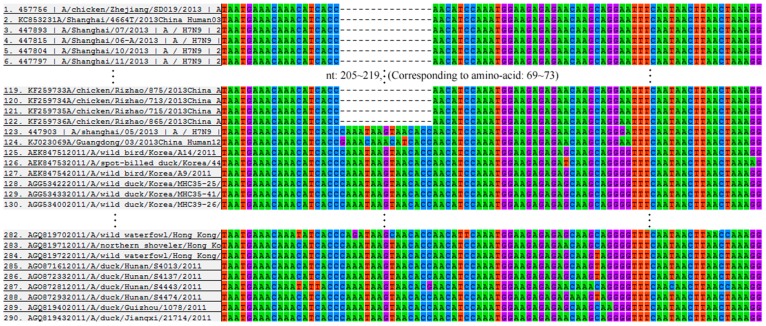
The alignment of N9 sequences affiliated to novel AIVs H7N9 and other subtypes. Only displayed the comparison between novel AIVs H7N9 and the isolates established during 1 January 2011–31 December 2011 in Asia. Sequences to be longer than 1000nt are adopted.

For a long time, haemagglutinin and polymerase subunit PB2 are believed to be decisive factors for host tropism of influenza virus, and neuraminidase (NA) to be responsible for virulence and interaction with drugs [5,6]. In the current analysis of neuraminidase of N9 subtype, we identified a deletion of amino-acids 69 to 73 in the neuraminidase stalk (Figure 1). This was the notable characteristic of the novel AIV H7N9, which might have strengthened its human tropism.

## 2. Material and Methods

### 2.1. Data Sources

Neuraminidase gene sequences of N9 subtype were obtained from NCBI Influenza Virus Sequence Database and the Global Initiative on Sharing Avian Influenza Database (GISAID) (Time & Date, GMT + 8, 10:00, 18 May 2014) [7,8]. Sequences were discarded if: (1) they were the same sequences deposited in NCBI and GISAID simultaneously; (2) they were too short to cover nucleotide sites 205–219 nt that corresponded to 69~73 aa in neuraminidase protein; and (3) the hosts of the sequences could not be tracked.

### 2.2. Analysis

MEGA6.0 software was used for sequence aligning and phylogenetic tree constructing; after W method alignment, a phylogenetic tree was constructed by p-distance. Chi-square test in Stata12.0 software were used for analysis of significance. UCSF Chimera was employed for protein tertiary structure homology modeling. As in the procedure of protein homology modeling, according to Li *et al.* and Xiong *et al.* [1,2], the representative novel AIV H7N9 and its two possible ancestors were A/Zhejiang/DTID-ZJU01/2013 (H7N9) (Accession No. KC885958), A/wild bird/Korea/A14/2011 (H7N9) (Accession No. JN244222), and A/Baikal teal/Hongze/14/2005 (H11N9) (Accession No. GQ184333) respectively. The PDB IDs of multi-model were 1NMB (X-ray diffraction), 3TIA (X-ray diffraction), and 4B7N (X-ray diffraction).

## 3. Results and Discussion

### 3.1. Results

#### 3.1.1. Statistical Analysis

A total of 736 neuraminidase gene sequences of N9 subtype were obtained, including H1N9 (53), H2N9 (54), H3N9 (8), H4N9 (25), H5N9 (19), H6N9 (13), H7N9 (173), H8N9 (0), H9N9 (10), H10N9 (13), H11N9 (306), H12N9 (4), H13N9 (19), H14N9 (0), H15N9 (6), H16N9 (1), H17N9 (0), H18N9 (0), and mixed HA (32). Of these H7N9 subtype consisted of 124 novel isolates established in 2013 and 49 established beforehand. There were 81 human isolates of the novel AIV H7N9, of which 79 had a deletion of amino-acids 69 to 73 in the neuraminidase stalk. Among all the 654 avian origin sequences however, only 43 had the deletion (the other, A/whale/Maine/1/1984 (H13N9), is a whale origin H13N9 established in 1984). The difference in the percentage of this deletion was statistically significant (*p* < 0.001).

#### 3.1.2. Phylogenetic Tree Construction

**Figure 2:**
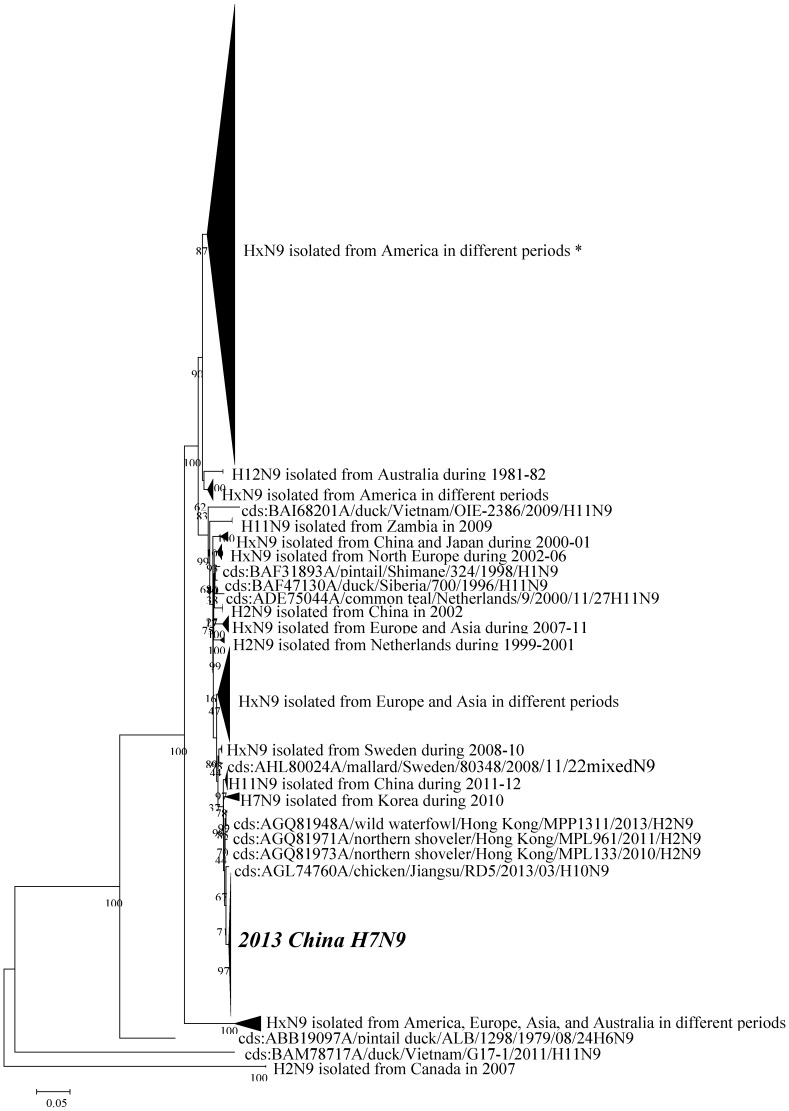
Phylogenetic tree of neuraminidase gene sequences of N9 subtype influenza A viruses. It indicated that the novel H7N9 AIVs isolated in China 2013, whether or not they were characterized by the deletion of amino-acids 69 to 73 in the neuraminidase stalk, shared very close homogeneity. ^*^ Note, x such as in HxN9 indicated that there was more than one subtype HA in the clade.

Phylogenetic tree displayed that the neuraminidase gene sequences affiliated to the novel H7N9 AIVs isolated in China 2013 shared very close homogeneity, whether or not they were characterized by the deletion of amino-acids 69 to 73 in the neuraminidase stalk. It also revealed that sorts of N9 subtype influenza A viruses had a remarkable tendency to cluster according to their geographical distributions (Figure 2).

#### 3.1.3. Homology Modeling

**Figure 3:**
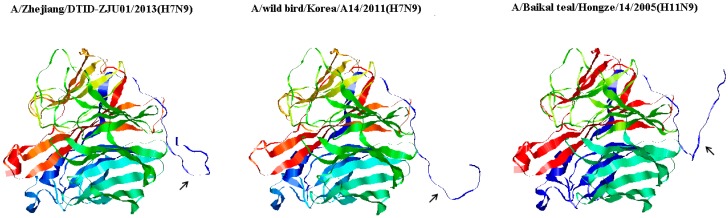
Tertiary structures of neuraminidase affiliated to novel AIV H7N9 and its ancestors. Compared with two ancestors, novel AIV H7N9 with deletion in its neuraminidase stalk had a more compact spatial structure. The deletion and its corresponding regions in neuraminidase stalk of novel AIV H7N9 and its ancestors are pointed by arrows.

Homology modeling for neuraminidase displayed that the deletion obviously changed its spatial direction, and resulted in a more compact neuraminidase structure in the novel AIV H7N9 (Figure 3).

### 3.2. Discussion

The host range of influenza A virus is determined by amino acid residue substitutions or receptor binding specificity. The HA molecule primarily contributes to the determination of host range by receptor specificity [9]. In contrast, internal viral proteins such as polymerases are required to replicate the viral genome in the host cell. Hiroichi *et al.* generated 7 + 1 reassortant viruses in which one genome segment derived from A/chicken/Hong Kong/W312/97 (H6N1) virus was replaced with that of A/duck/Shantou/5540/01 (H6N2) isolated from wild aquatic birds and they found that chimeras of PB2 and M genes, encoding the *C*-terminal region of the PB2 protein and M2 protein from W312 were required for efficient replication in canine-derived (MDCK) cells and in chicken trachea. These results indicate that host range may be determined by some types of internal proteins such as PB2 and M2, as well as by surface glycoproteins like hemagglutinin [10]. Other factors such as the influenza-induced “cytokine storm” at the site of infection in humans, together with the autophagy mechanism that might take place in cells under metabolic stress, through which cells can recycle waste material, could contribute to the severity of infection of influenza A virus. But it was rarely reported that mutations in the neuraminidase stalk could represent a determining factor in the tropism of influenza virus [11,12]. Based on series of analyses, we put forward the hypothesis that the deletion in neuraminidase stalk of N9 subtype could have strengthened human tropism of the novel AIV H7N9, as well as its virulence.

N9 subtype influenza virus was first found in Canada in 1966 and later in USA in 1968. There were no deletion in neuraminidase stalk and no spillover to humans until the 2013 epidemic in China. Possible impact of the deletion in N9 subtype AIV has not been previously reported, but deletions in N1, especially H5N1, could significantly influence AIVs’ host species specificity. Matsuoka *et al.* observed that among 162 NA sequences of human H5N1 isolates from 1996 to 2007, only two viruses isolated in Hong Kong 2003 contained complete length stalk NA. The short-stalk NA of H5N1 viruses circulating in Asia were conceived to be of adaptability in human infection [13]. Furthermore, there was a ratio increase of such special NA stalk motifs in human derived H5N1 isolates from 2000 to 2007, it was only observed in two of the five total strains in 2003, but it was observed in all 173 strains from 2004 to 2007 [14]. The H5N1 viruses isolated from other mammalian hosts, including pigs, cats, leopards, tigers, and dogs, also had short NA stalks [13,15]. Upon transmission of avian influenza viruses from waterfowl to domestic birds, deletions in the NA stalk region have also been detected frequently [16]. Up to now, stalk deleted NAs with assorted motifs were reported sporadically in several AIV subtypes other than H7N9, e.g., H5N1, H6N1, H7N1, H7N3 and H9N2, and are often accompanied by observations about variants on host range [17,18]. Series of signs indicate that NA may act as another tropism associated surface glycoprotein in influenza virus; occurrence deletion in the stalk, as well as the length of deletion, to a large extent, are pivotal factors to determine which host spices can be infected by the virus.

NA stalk length also affects other biological properties of influenza viruses. Both Zhou *et al.* and Matsuoka *et al.* above-mentioned found that virulence in mice was enhanced for viruses with a truncated NA stalk compared to their equivalents with a long stalk. Viruses with a short-stalk NA showed a decreased capacity to elute from red blood cells and an increased virulence in mice [13,14]. Data of Munier *et al.* indicates that a shortened NA stalk is a strong determinant of virulence and adaptation of waterfowl influenza viruses in chickens [16]. Possible mechanism is that the deletion in neuraminidase stalk results in a more compact neuraminidase structure. However, a stretched stalk could make neuraminidase have more accessibility to the substrates (*i.e.*, 2–6 or 2–3 glycosides) around it, which subsequently results in more substrates cleaving and less novel infecting, in return less virulence [19,20]. In view of the fact that the novel AIV H7N9 had a much higher fatality rate compared with pandemic influenza H1N1, a deletion in its neuraminidase stalk could have a similar effect.

Considering subtypes of AIVs carried by poultry in eastern and southern China and its possible reassortments [21,22], epidemiological and virological surveillance for this novel N9 with a deletion in its stalk should be further strengthened.

## 4. Conclusions

Although there is no experimental proof, statistical significance highlights the fact that there exists a remarkable difference in the host tropism of AIVs H7N9 with or without the deletion of amino-acids 69 to 73 in the neuraminidase stalk; tertiary structure homology modeling displays that the deletion notably changed the spatial direction of neuraminidase. We hence put forward an opinion that the deletion in neuraminidase stalk could have strengthened human tropism of the novel AIV H7N9, as well as its virulence. The results of this study should be interpreted with caution due to the limitations identified. And thus, further research warrants more experimental proof for these conclusions.
